# Numerical Simulation and Experimental Verification of Residual Stress in the Welded Joints of Weldolet–Branch Pipe Dissimilar Steels

**DOI:** 10.3390/ma15031044

**Published:** 2022-01-28

**Authors:** Chunliang Mai, Xue Hu, Lixin Zhang, Bao Song, Xiongfei Zheng

**Affiliations:** 1 College of Mechanical and Electrical Engineering, Shihezi University, Shihezi 832003, China; maichunliang@stu.shzu.edu.cn (C.M.); zhlx2001329@163.com (L.Z.); songbao@hust.edu.cn (B.S.); zxf13821795839@163.com (X.Z.); 2 College of Mechanical Science and Engineering, Huazhong University of Science and Technology, Wuhan 430074, China

**Keywords:** weldolet, branch pipe, interlayer temperature, welding sequence

## Abstract

It is well known that welding dissimilar metals can play the advantages and characteristics of those different metals, but it is easy to encounter some problems. In this paper, the thermomechanical behavior of the weldolet–branch dissimilar steel joints in different welding cases is analyzed by establishing a three-dimensional finite element model, and the predicted thermal cycling and residual stresses are verified using experimental tools. The results show that the high temperature area and the heat affected zone on the side of the branch pipe are larger, and there is a large stress gradient at the fusion line on both sides of the weld. Too high or too low temperature between welding layers will cause large residual stress, thus, 200 °C is more suitable for the welding of weldolet–branch joints. The residual stresses of path-1, path-2 and path-3 have similar distributions at 0° and 180° sections, and the circumferential and axial residual stresses on the inner surface are larger than those on the outer surface. The residual stress on the inner and outer surfaces of path-3 is smaller than that of path-1 and path-2 at the 90° and 270° sections as a whole, and the residual stress at the 90° section reaches the minimum.

## 1. Introduction

As a new type of reinforcing receiver, the weldolet is widely used in pipeline systems of chemical plants, thermal power plants and nuclear power plants. The weldolet can directly replace the traditionally used branch pipe connections, such as reducer tees and reinforced pipe sections, with higher safety and economy. It is especially used more frequently in high temperature, high pressure and even ultra-high pressure and subcritical pipelines [1,2,3,4,5]. With the increasing industrialization all over the world, various new structures and equipment are emerging in an endless stream, the application of new materials and new processes is becoming more and more extensive, and the research on various new materials and composite structures is becoming more and more extensive and in-depth. The single material in traditional industrial manufacturing can no longer meet the needs of various industries, which forces the manufacturing industry to develop in the direction of lighter structure, multi-function and low cost [6,7]. In general, the realization of the interconnection of dissimilar materials can combine the advantages of these materials and compensate for the defects of different materials themselves, which, to some extent, effectively meets the production requirements [8,9,10].

In the traditional manufacturing field, 12Cr1MoV and 15CrMo are both indispensable engineering materials. Both 12Cr1MoV steel and 15CrMo steel are low-alloy pearlitic heat-resistant steels, which are widely used in high-temperature and high-pressure steam pipes and superheater pipes due to their good oxidation resistance, high strength and plasticity, simple production process and high lasting strength at higher temperatures [11,12,13]. In practical engineering, we often encounter problems with the welding of two types of steel. Compared to the welding of the same material, welding between dissimilar materials is much more difficult. The physical properties and chemical composition of the metal itself limit the welding quality, and the difference in the welding performance of the two metals will affect the weldability between them to a greater extent [14,15,16,17]. Therefore, in order to achieve a high-efficiency and high-quality connection between 12Cr1MoV and 15CrMo, it is necessary to choose a reasonable welding method, formulate a reasonable welding process and ensure the quality and mechanical properties of the weld joint.

Since the advent of the thermal-elastoplastic finite element model, numerical simulation research on welding stress and deformation of the same type of steel and dissimilar steel has been developed rapidly. The welding finite element modeling process mainly includes moving heat sources, temperature-related material thermophysical properties, thermal and mechanical analysis, etc. [18]. The most important component in the finite element modeling process of dissimilar steel is to give different material performance parameters to different welded components, and these thermophysical performance parameters all change with temperature. Trupiano et al. [19] introduced a new equivalent parametric modeling method for simulating longitudinal multi-pass welds on planar structures such as slabs and rectangular hollow sections. The proposed Welding Equivalent Model (WEM) employs single-layer and multi-layer shell, link and beam elements. It helps detect residual stresses and local deformations typical of multi-pass welds. Lee et al. [20] predicted the temperature field and residual stress state in welded joints of carbon and stainless steels based on the finite element method, and the results showed that the weld residual stresses in dissimilar steel butt welds are different from those in corresponding similar steel butt welds. Huang et al. [21] used SYSWELD software to carry out numerical simulation and experimental verification of the temperature field, HAZ microstructure evolution, residual stress and post-weld deformation of S355JR-316L dissimilar metals. Sauraw et al. [22] achieved the joining of P91 and P22 steels using a gas tungsten arc welding (GTAW) process, and measured the microstructural inhomogeneity and element diffusion across the interface of the welded joint. At the same time, the mechanical properties of the welded joints under as-welded and post-weld heat treatment conditions were evaluated on the basis of experiments. Sepe et al. [23] performed finite element simulations and experimental tests on dissimilar steel T-joints, which were analyzed in detail with respect to the temperature distribution during welding and the deformation at the end of final cooling. Khamari et al. [24] compared and analyzed the mechanical and microstructural properties of plates with different thicknesses welded by gas tungsten arc welding (GTAW) and electrode arc welding (SMAW), and designed a new set of welding parameters for structural steel welding. Karalis et al. [25] investigated the effect of the thermal uncertainty encountered during shielded metal arc welding (SMAW) of thin welded plates made of Ck45 steel on the out-of-plane corner deformation of the welded panels by means of the finite element method and experimental measurements. Taghipour et al. [26] observed the microstructure of the pipe–flange welded joint fracture and combined it with the simulation. The study found that the weld bead area at the root of the welded joint is composed of large grain structures, and the hardness of the weld root area is low. The reason for the fracture is unreasonable welding parameters. Xia et al. [27] simulated the multi-layer welding process of dissimilar welded joints through thermal–mechanical coupling analysis. The results show that, due to the different material properties, the residual stress in different pipe joints is asymmetric. Due to the welding sequence and the welding start/stop position, the residual stress distribution is very different. The change in residual stress on both sides of the weld centerline is more complicated than that of similar welded joints. Pamnani et al. [28] used finite element model (FEM) simulation to study the thermodynamics of DMR-249A steel welds fabricated by shielded metal arc welding (SMAW) and active gas tungsten arc welding (A-GTAW) processes’ properties; the results of the study demonstrate the applicability of the numerical model to be an effective method for predicting the thermomechanical properties of DMR-249A steel welded joints affected by welding techniques. Li et al. [29] designed a 5%Cr weld metal for 9Cr/2.25Cr dissimilar weld joints. Studies have shown that the alloy design of 5%Cr weld metal has a significant effect on reducing the degree of carbon migration at the joint interface. The interface creep damage and early failure tendency are greatly reduced. Li et al. [30] showed that the solid-state phase transformation of P92 steel, the strain-hardening and annealing effects of SUS304 stainless steel and the difference in thermal expansion coefficients between the different metals have a significant effect on the formation of longitudinal residual stresses in P92/SUS304 butt welds, and that X-groove butt welds may be superior to single V-groove butt welds in reducing areas of high residual tensile stresses. The current research on dissimilar steel welding is mainly focused on flat joints, T-pipe joints or pipe plate joints, etc. However, there is almost no research for the more special structure of the weldolet. There are two types of welds in the complete weldolet welding process. One end of the weldolet is a saddle weld, and the other end is a circumferential weld. The distance between the two welds is very close. Therefore, strict control of the welding quality of the circumferential weld between the weldolet and the branch pipe is also very important for the overall safety of the weldolet. Weldolets are commonly used in the piping system of thermal power plants, the base material is mostly 12Cr1MoV, the branch pipe is mostly 15CrMo and the welding characteristics of the two materials are also relatively rare. At the same time, previous research scholars for dissimilar steel welded joints focus on the analysis of the interface macroscopic structure of the joint and the mechanical properties of the joint, or they just study the temperature and residual stress distribution characteristics of dissimilar steel joints, while the different welding process parameters, such as the welding sequence and interlayer temperature, for dissimilar steel joints’ temperature and residual stress distribution are less commonly researched. Therefore, this paper considers both the interlayer temperature and the welding sequence to carry out the welding process optimization study of the weldolet–branch dissimilar steel welded joints.

In this study, the hexahedral mesh model was used to simulate and calculate the welding process of the weldolet–branch pipe dissimilar steel annular weld and verify the feasibility of the calculation method by means of experimental analysis. By comparing and analyzing the arc welding process of 12Cr1MoV heat-resistant steel and 15CrMo heat-resistant steel under nine welding process parameters, the effect of process parameters on the thermal cycle and residual stress of dissimilar steel joints was revealed, which is of great significance for the selection and optimization of the process.

## 2. Experimental Procedure

**Figure 1:**
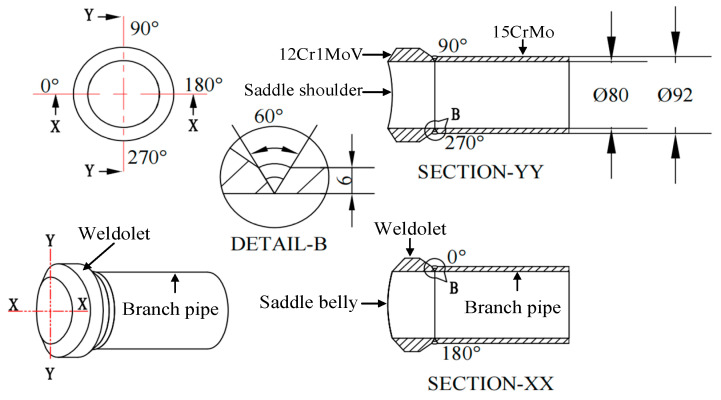
Two-dimensional (2D) diagram of the weldolet–branch pipe structure.

**Figure 2:**
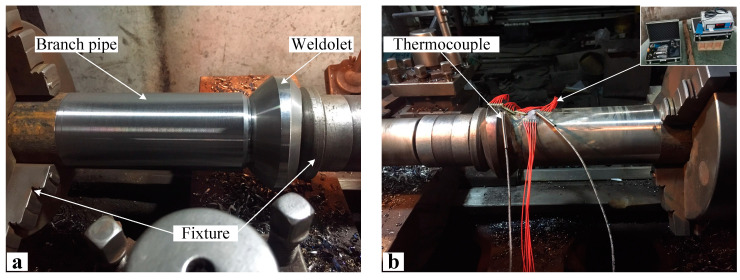
The welding experiment setup of the weldolet–branch pipe (**a**) before welding and (**b**) after welding.

The experiment object is weldolet–branch pipe dissimilar steel welding. The size of the weldolet is DN80 × 16 mm, and the material is 12CrMoV steel. The size of the branch pipe is DN80 × 6 mm, and the material is 15CrMo steel. The selection of welding materials for dissimilar steel welded joints uses low matching, so the filler metal matching with 15CrMo is selected. That is, tungsten inert gas welding (GTAW) using H08CrMoA (China, Guangzhou)heat resistant steel argon arc welding wire and electrode arc welding (SMWA) welding using R307 electrode. The sizes of the welded joints, the bevel angles and the distribution of the weld channels are shown in Figure 1. The weldolet–branch pipe welding adopts a single-sided V-shaped groove. The weldolet is a machined standard part with its own bevel, and the bevel angle is 30°. The branch pipe is machined and also has a 30° bevel. According to the technical regulations for welding of dissimilar steels in thermal power plants, the interlayer temperature is selected as 12Cr1MoV with a higher preheating temperature of the parent material, set at 200 °C, and the interlayer temperature and preheating temperature of the welding process should be consistent. Before welding, spot weld the measuring ends of the thermocouple on the outer surface of the weldolet side and the branch pipe side, respectively, and the distance between the measuring point and the center line of the weld is 16.5 mm. The other end of the thermocouple is connected to the input end of the temperature measuring instrument, and the temperature value during the welding process is recorded in real time. Figure 2 shows the setup of the weldolet–branch pipe welding experiment.

During the welding process, no external constraints are imposed on the components, so the components can move freely in different directions. Before welding, the surrounding points of the butt joint of the weldolet and the branch pipe are fixed by spot welding. The rust, oil and other impurities near the groove are cleaned up before welding. The experimental process was a two-layer, two-pass weld, and the welding sequence was a single-pass straight-through type with GTAW priming and SWAM filler capping. The shielding gas is pure argon, and the welding parameters of the different welding channels are shown in Table 1. The workpiece needs to be preheated before welding, and the preheating temperature and the interlayer temperature are kept the same.

## 3. Finite Element Analysis

The rapid professional welding software, Simufact Welding 2020, was used to simulate the welding temperature field and residual stress field of the dissimilar steel welded joints. The algorithm of this software is based on the thermo-elastic–plastic finite element model, which can conveniently prepare input data according to welding conditions, welding seam arrangement and welding sequence [31,32]. The thermal–mechanical behavior was simulated using the sequential thermodynamic coupling method during the welding process. The calculation method consists of two steps. The first step is the thermal analysis. In this step, the temperature history of all nodes involved in the finite element model is calculated based on the welding conditions and thermal boundary conditions. The second step is the mechanical analysis. In this step, the thermal cycles of all nodes calculated in the first step are used as thermal loads. In the actual welding experiment, the flame gun is used to preheat and ensure the interlayer temperature. In the numerical simulation, preheating is performed by setting the initial temperature of different components, and the interlayer temperature is controlled by setting the start–stop time of the welding torch.

### 3.1. Finite Element Modeling

**Figure 3:**
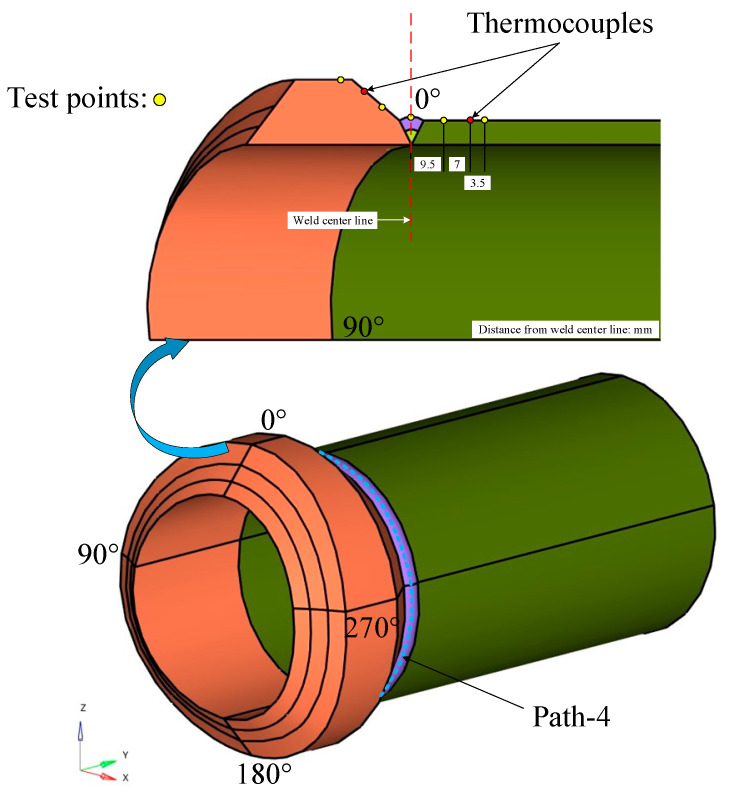
Three-dimensional (3D) model of the weldolet and branch pipe.

**Figure 4:**
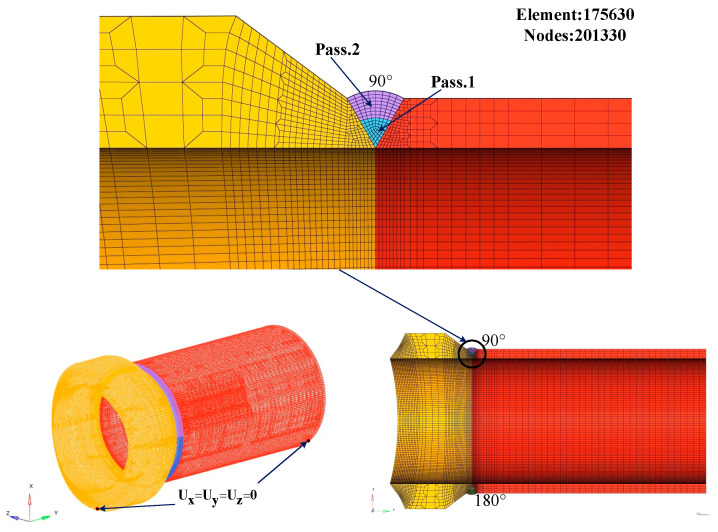
Finite element model meshing and boundary condition.

The three-dimensional finite element model of the weldolet, branch pipe and weld channel distribution is shown in Figure 3, and the weld channel is divided into the priming layer and the filling cover layer. The mesh division adopts the professional mesh division software Hypermesh 2019. The most important part of the meshing is to cut the model into fusion zone, heat affected zone and other areas. The 2D meshing is performed first, and, finally, the 3D mesh is generated with the mapping command. In order to improve the computational efficiency and ensure the accuracy of the numerical simulation, the mesh of the weld and the nearby area is refined, and the mesh of the area far from the weld is gradually coarsened. The number of elements in the simulation model is 175,630, and the number of nodes is 201,330. Figure 4 shows the finite element meshing of the weldment.

### 3.2. Heat Source Model and Thermal Analysis

In the welding process, the governing equation of heat transfer is as follows:

(1) $$\rho c\frac{\partial T}{\partial_{t}}=\frac{\partial }{\partial_{x}}(\lambda \frac{\partial T}{\partial x})+\frac{\partial }{\partial y}(\lambda \frac{\partial T}{\partial y})+\frac{\partial }{\partial_{z}}(\lambda \frac{\partial T}{\partial_{z}})+\bar{Q}$$

where c is the specific heat capacity of the material, ρ is the density of the material, T is the temperature, t is the time, λ is the thermal conductivity of the material and $\bar{Q}$ is the intensity of the internal heat source.

**Figure 5:**
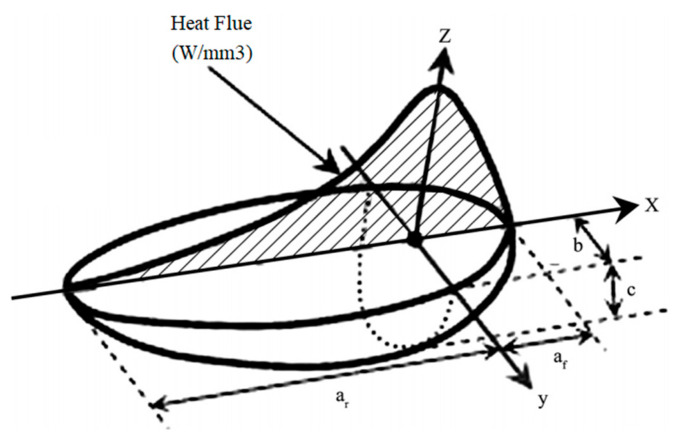
Double ellipsoid heat source model.

The heat source model plays a crucial role in thermal and mechanical analysis. In this study, the double ellipsoidal heat source model proposed by Goldak et al. [33] was used. Adjusting the heat source parameters according to the actual molten pool shape ensures the calculation accuracy of welding residual stress. The heat flux density in the first half and the second half of the heat source model can be described by Equations (2) and (3).

(2) $$q_{f}\left(x,y,z\right)=\frac{6\sqrt{3}f_{f}Q}{abc_{f}\pi \sqrt{\pi }}\exp\left(-\frac{3x^{2}}{c_{f}^{2}}-\frac{3y^{2}}{b}-\frac{3z^{2}}{a^{2}}\right)$$

(3) $$q_{r}\left(x,y,z\right)=\frac{6\sqrt{3}f_{r}Q}{abc_{r}\pi \sqrt{\pi }}\exp\left(-\frac{3x^{2}}{c_{r}^{2}}-\frac{3y^{2}}{b^{2}}-\frac{3z^{2}}{a^{2}}\right)$$

where Q is the heat source power, $a,b,c_{f}\;\mathrm{and}\;c_{r}$ are the ellipsoidal parameters, $f_{f}$ and $f_{r}$ are the heat distribution of the front and rear ellipsoids, respectively, and $f_{f}+f_{r}=2$. Q= ηUI, U is the welding voltage, I is the welding current and η is the arc efficient. In this study, η was assumed to be 0.7. The double ellipsoidal heat source model is shown in Figure 5.

**Figure 6:**
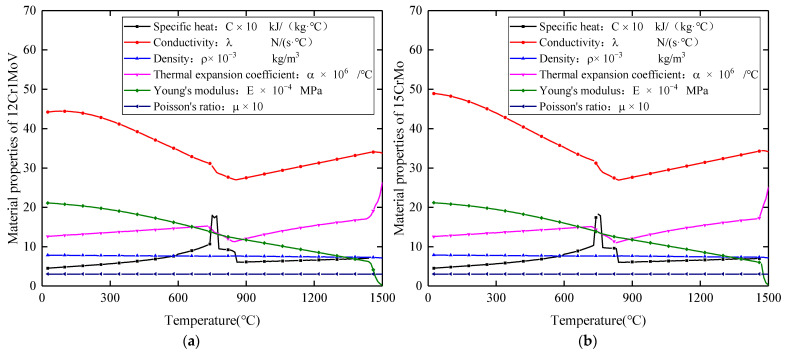
Temperature-dependent thermodynamic properties of (**a**) 12Cr1MoV steel and (**b**) 15CrMo steel.

In the process of numerical simulation of welding, the thermophysical properties of the material parameters have a significant impact on the calculation results. The thermodynamic properties of 12Cr1MoV and 15CrMo change with temperature; the relevant thermophysical property parameters of the two base materials used in the paper were obtained through JMatPro professional software. The thermomechanical properties of the two base materials are shown in Figure 6 and Table 2. The weld filling process uses the “living and dead element method”, where when the heat source is activated in the area of the element, the heat source does not reach the area of the weld element in an inactive state. With the movement of the welding heat source, the weld material is filled into the weld in turn.

The finite element model of this paper considers the heat loss in welding, and the heat exchange between the weldment and the environment includes two forms: convection heat dissipation and radiation heat dissipation. Heat loss is described by Newton’s law (4) and Stephen–Boltzmann’s law (5).

(4) $$Q_{c}=h\left(T-T_{0}\right)$$

(5) $$Q_{r}=\varepsilon \sigma \left(T^{4}-T_{0}^{4}\right)$$

where *h* is the heat transfer coefficient ($20{\;W\;m}^{-2}\cdot K^{-2}$), T is the surface temperature of the weldments, $T_{0}$ is the ambient temperature (25 °C) and ε is the thermal emissivity. Given 0.6, σ is Stefan–Boltzman constant.

### 3.3. Mechanical Analysis

In the mechanical analysis, the results of the thermal analysis are loaded into the elastic–plastic analysis as thermal loads, and the total strain of the material in the finite element model can be described by the following equation:

(6) $$\varepsilon^{total}=\varepsilon^{e}+\varepsilon^{p}+\varepsilon^{th}+\varepsilon^{tr}\;$$

where $\varepsilon^{total}$ indicates total strain, $\varepsilon^{e}$ indicates elastic strain, $\varepsilon^{p}$ indicates plastic strain, $\varepsilon^{th}$ indicates thermo-metallurgical strain and $\varepsilon^{tr}$ indicates phase change strain. For high carbon steels, the solid–solid transformation has a considerable effect on the mechanical behavior. The strain caused by the phase transformation mainly considers the effect of the volume and yield strength changes caused by the austenite–martensite transformation on the welding residual stress. In addition, the effect of creep-induced strain on the total strain can be neglected due to the material stays at high temperatures for a very short time during the whole process. The elastic strain components were modeled using isotropic Hooke’s law with temperature-dependent Young’s modulus and Poisson’s ratio. The plastic behavior was modeled using the von Mises criterion, temperature-dependent mechanical properties and isotropic hardening. The thermo-metallurgical strain is considered the strain due to the temperature-dependent thermal expansion coefficient and phase change. The finite element model was set up with experimentally consistent boundary conditions, and the experimental data were used to validate the weld simulation results. These include the assumption that the weldolet–branch pipe is free to deform in any direction without external constraints, but rigid body motion in all directions is prevented. The boundary conditions of the finite element model are Ux=Uy=Uz=0 in Figure 4, which are located at both ends of the weldolet and the branch pipe, respectively. In the welding experiment, not only the fixture was set, but also the two models were fixed by spot welding. The position of spot welding is mainly at four symmetrical points, but the heat input of spot welding is very small, and its influence on welding residual stress can be ignored. Therefore, the boundary conditions matching the fixture are set in the finite element simulation process, and the influence of spot welding is not considered.

### 3.4. Simulation Cases

**Figure 7:**
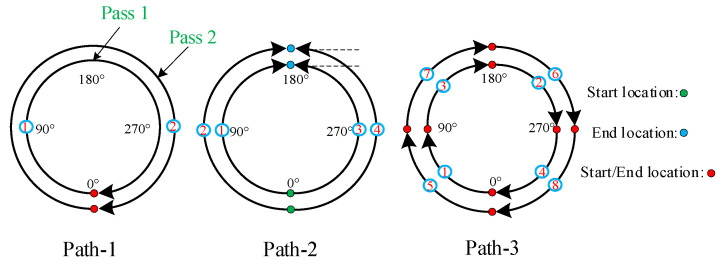
Three welding sequence settings.

Figure 7 shows the three welding sequences proposed during the numerical simulation. Welding sequence path-1 is the continuous welding of two welds in a clockwise circle. Each layer of weld starts from the 0 position, then proceeds in the clockwise direction, and finally ends at the 360° (0°) position. Welding sequence path-2 is a two-part weld, with each full-cycle pass divided into two parts. The first part is a two-layer weld path of 0°–90°–180°. The arc of the first layer of welds is clockwise from position 0° to position 180°, and then the second layer of welds remains clockwise from position 0° to 180°. The second part is a 0°–270°–180° weld, with each layer of welds going counterclockwise from the 0° position to the 180°. In the welding sequence path-3, each full-circle weld is divided into four parts. The welding process adopts segmented symmetrical jump welding, and the welding direction is clockwise. The arrows in the figure indicate the welding directions, and the numerals indicate the welding steps.

The finite element model in this paper is a multi-layer multi-pass welding, which needs to control the temperature between layers reasonably. Therefore, three groups of interlayer temperatures were set up for numerical simulation, and the interlayer temperatures suitable for this model were analyzed. The welding case of this numerical simulation can be obtained by permutation and combination of the two welding factors, as shown in Table 3.

## 4. Results and Discussion

### 4.1. Comparison of Experimental Result and Numerical Simulations

**Figure 8:**
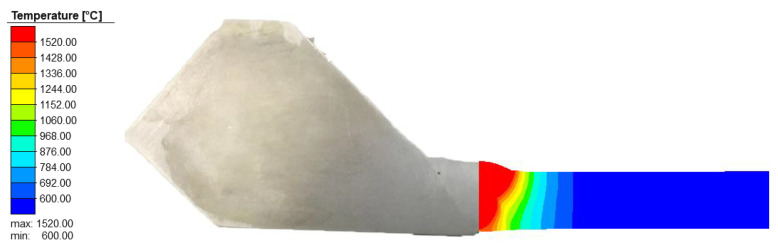
The shape of the weld pool obtained through experiments and finite element simulations.

Obtaining a reasonable temperature field of welded joints is the prerequisite for the analysis of welding residual stress. In order to accurately simulate the shape of the weld pool and ensure the accuracy of the calculation of the weld residual stress, based on the thermal-elastic–plastic finite element model, the heat conduction equations are solved using a multi-threaded parallel technique. Eight threads were called simultaneously during the temperature field calculation. The calculated transient melt pool shape and the macroscopic shape of the experimental joint are shown in Figure 8. It can be seen that the temperature of the fusion zone is above 1520 °C, and the area of the transient heat source’s molten pool is almost equal to the area of the weld channel. The calculated results of the weld molten pool are in good agreement with the experimental results, indicating that the calculated results of the temperature field are valid and reasonable.

In order to verify the prediction accuracy of the developed calculation method, we compared the experimental measurement results of transient temperature and residual stress with the finite element simulation results. The k-type thermocouple is used to measure the transient temperature at different positions on the outer surface of the dissimilar steel welded joint. The position distribution of the thermocouples on the outer surface of the welded component is shown in Figure 3. They are located 16.5 mm from the centerline of the weld on the side of the weldolet and 16.5 mm from the centerline of the weld on the side of the branch. The residual stress is measured by blind hole method and stress measuring instrument. The location of the stress measurement points in the welding experiment is shown in Figure 3.

**Figure 9:**
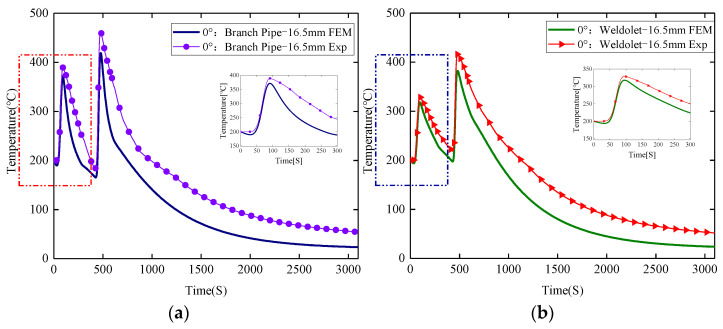
Comparison of predicted and measured values of transient temperature: (**a**) branch pipe side, (**b**) weldolet side.

It can be seen from Figure 9 that there is a certain difference between the transient temperature curve measured by the thermocouple and the numerical simulation calculation result. The peak temperature obtained by the experimental measurement is slightly higher than the numerical simulation calculation result, and the maximum difference is 43.5 °C. When the heat input is small, the heating and cooling phases of the thermal cycle curve are in good agreement, but as the heat input increases, the actual cooling rate in the welding process is slightly larger than the calculated value, but the overall trend is in good agreement. It is also seen that, although the measured two points located on both sides of the weld are at the same distance from the center of the weld, they have different peak temperatures, heating rates and cooling rates. This is caused by the different thermophysical property parameters of the different base materials. 15CrMo steel has a higher thermal conductivity and specific heat capacity coefficient, and therefore higher peak temperature and heating and cooling rates. From this, the verification results show that the established heat source model simulates the heat process of the weldolet–branch pipe dissimilar steel welding well, which lays the foundation for the stress calculation.

**Figure 10:**
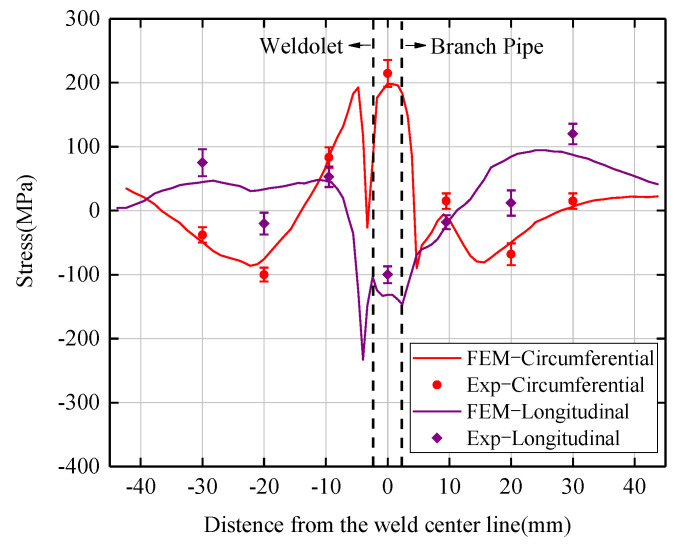
Comparison of the residual stress obtained from the experiment and the finite element calculation.

We measured the residual stresses at selected points on the outer surface of the weldolet and branch pipe, respectively, at 90° cross-section. The five measuring points are located at the centerline of the weld and at 9.5 mm and 20 mm from the centerline of the weld on both sides of the weld. The experimental results and finite element simulation results are shown in Figure 10 with error lines for the experimental data. It can be seen that the results are reasonably consistent with each other, except for the close matching of trends. For the existence of the difference, the reason is attributed to three aspects: the first is the existence of errors between the finite element model and the actual welded components. The second is that the position of the experimental measurement point and the mesh node of the corresponding point in the finite element model cannot be perfectly matched, because the volume mesh node in the finite element model is generated according to the model size and cannot be changed at will. The third is the numerical simulation of the finite element model set to the ideal state, that is, based on the actual welding process, set to match the welding process parameters, material thermodynamic physical properties parameters, boundary conditions, etc., but to ignore the residual stresses generated by the welding parts in the pre-processing process before welding residual stresses inside the welded parts. Based on the above analysis, the finite element calculation method established in this study can be used to study the temperature and residual stress distribution in the multi-layer multi-pass welded joint of the weldolet–branch pipe.

### 4.2. Simulation Results of Thermal Field

**Figure 11:**
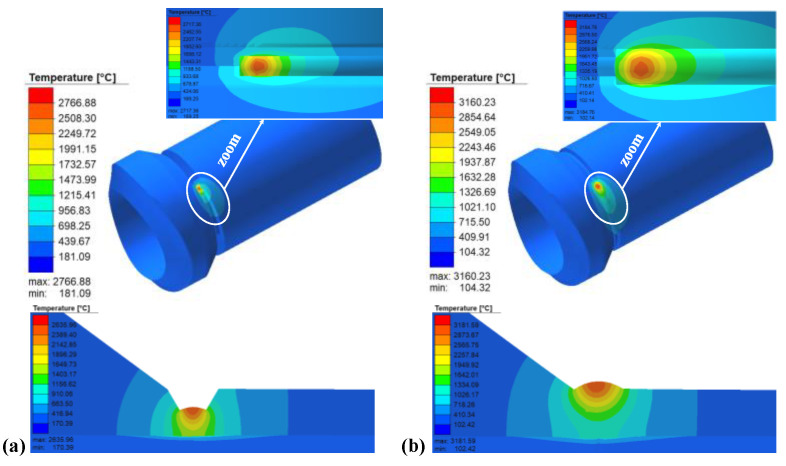
Numerically estimated thermal field: (**a**) first layer weld and its cross section, (**b**) second layer weld and its cross section.

**Figure 12:**
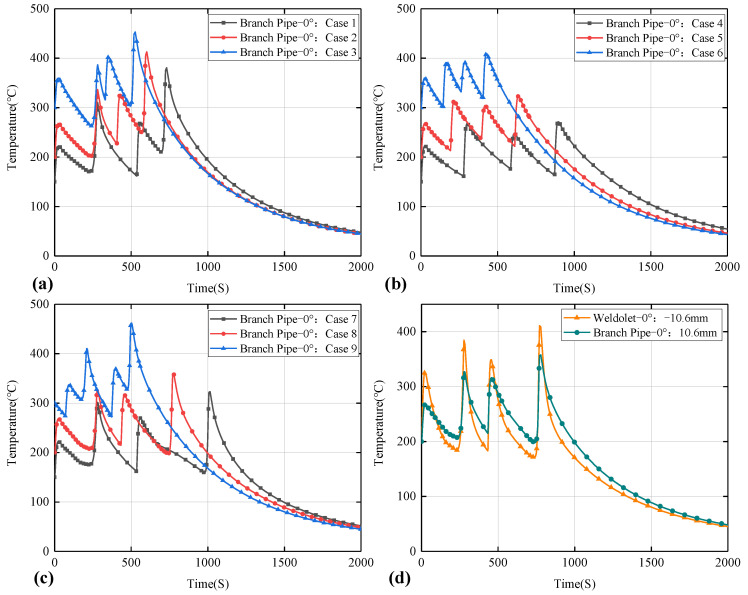
Temperature history curve (**a**) Case 1–3 (**b**) Case 4–6 (**c**) Case 7–9 (**d**) Thermal cycle comparison between the weldolet-side node and the branch-side node.

The thermal field of the weldolet–branch pipe structure is shown in Figure 11; the temperature at the center of the molten pool is much higher than the melting point of the weld metal by 1520 °C. Obviously, the shape of the elliptical melt pool in the weld is consistent with the double ellipsoidal heat source model. The distribution of isotherms is characterized by a dense front and a sparser rear. At the same time, the temperature field distribution on both sides of the weld centerline is not symmetrical, the heat affected zone on the 12Cr1MoV side is significantly narrower than that of 15CrMo and the high temperature area of 15CrMo is also wider. The reason is that the thermal conductivity of 15CrMo steel is higher than that of 12Cr1MoV steel, which makes the temperature diffusion rate and cooling rate faster on the 15CrMo side, resulting in a wider high temperature area and a larger heat-affected zone on the 15CrMo side. In order to further analyze the temperature change of dissimilar steel joints in the welding process, the node on the branch side of the welded components at the 0° position cross-section in different welding cases is selected from the center of the weld at 10.6 mm to draw the thermal cycle curve. In the weldolet side of the same selection from the center of the weld’s 10.6 mm node, comparative analysis of the weldolet side and branch side of the same distance from the centerline of the weld thermal cycling curve, as shown in Figure 12.

Figure 12a–c respectively show the temperature distribution on the outer surface of dissimilar steel joints at 0° section under nine different welding cases (Case 1–Case 9). It can be observed that all thermal cycling curves have four peaks and troughs, which is due to the fact that the 0° position is both the weld start and weld end point in the three welding sequences. Due to the difference in temperature between welding layers, the peak temperature and the trough temperature are kept within a specific temperature range. Due to the different welding sequence, the time node of the peak appearance is also different. As the heat input increases, the peak temperature also increases. Figure 12d shows the temperature distribution on the side of the weldolet and the side of the branch pipe. It can be seen that although the distance from the center of the weld is the same, their peak temperatures and cooling rates are different. The peak temperature on the branch pipe side is lower, but the cooling rate is significantly higher than on the weldolet side. This is consistent with the conclusions obtained above. The characteristics of the thermal cycle curve change are very consistent with the characteristics of dissimilar steel welding. That is, the area of the heat-affected zone and the temperature conduction rate on the 15CrMo side are higher.

### 4.3. Welding Residual Stress along a Defined Path

The purpose of this study is to investigate the influence of the interlayer temperature and welding sequence on the residual stress distribution of dissimilar steel joints. In order to study the difference of residual stress distribution under nine cases, we analyzed the residual stress of the defined path-4 (shown in Figure 3). Path-4 is the centerline of the second weld surface.

**Figure 13:**
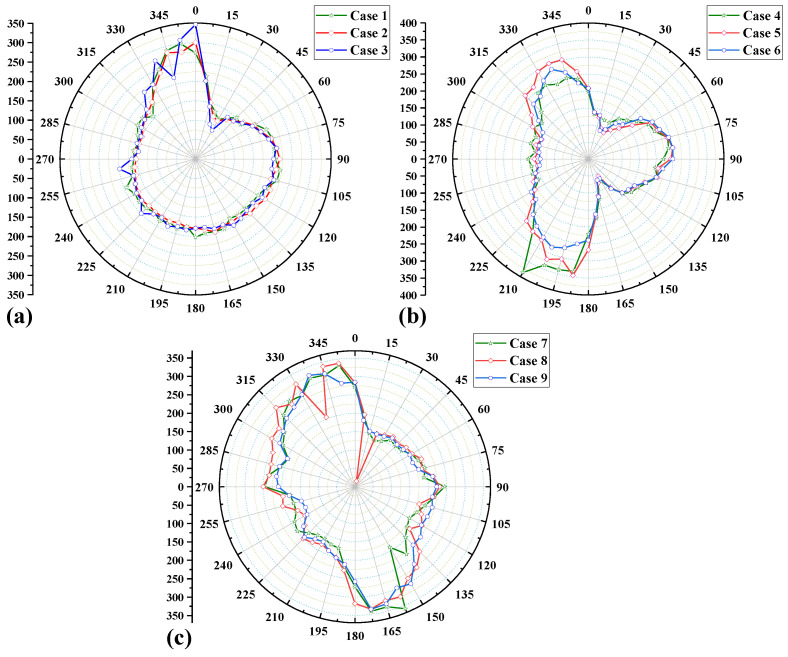
Circumferential residual stress distribution along path-4 for different welding cases. (**a**) Case 1–3 (welding sequence path-1) (**b**) Case 4–6 (welding sequence path-2) (**c**) Case 7–9 (welding sequence path-3).

Figure 13 compares the circumferential residual stress distribution along defined path-4 under nine welding cases. It can be seen from Figure 13a that the circumferential residual stress distribution of Case 1–3 (path-1) is very similar, because the welding sequence path-1 is used in these three cases, and the cladding sequence of the weld is the same. Comparing Case 1–3 carefully, we can find that the main difference between the three welding cases is the peak of the circumferential residual stress, which reaches a maximum value of 350 MPa at 0° (welding start position) in Case 3 (interlayer temperature of 300 °C). The circumferential residual stresses in Case 1 and Case 2 also reach a maximum around 0°, but the peak residual stress is slightly smaller than in Case 3. This is mainly due to the change in interlayer temperature of the weld. At the same time, we can find that for the welding sequence path-1, the circumferential residual stress at the position from 0° to 30° has a sudden change, and the circumferential residual stress at the position from 315° to 0° is significantly higher than other positions. The reason is that the 0° position is not only the starting point of the weld or the end point of the weld; it has experienced four thermal cycles, and multiple instantaneous heating and cooling instances lead to large plastic deformation of the metal material, so there is a large internal circumferential residual stress. Figure 13a also shows that within the range of ±30° on the left and right sides of the weld starting point at 0°, the circumferential residual stress on the left is much larger than that on the right. This is because the left side weld belongs to the post-welded side, while the right side belongs to the first side. The welding heat generated during the welding of the later side reduces the circumferential residual stress inside the weld on the first side.

Figure 13b shows the distribution of the circumferential residual stresses along the defined path for Case 4–6 (path-2). The maximum value of the circumferential residual stress is 326 MPa, which occurs at 210° in Case 4 (interlayer temperature of 150 °C), and also at the end of the weld on the back side. In addition, the high stress areas in Case 4–6 are mainly distributed in the 180°–240° area position and 315°–360° (0°) area position, which is mainly due to the fact that these two areas belong to the start and end of the weld position on the back weld side in path-2. This conclusion is very similar to the circumferential residual stress distribution characteristics of Cases 1–3, above. In contrast, the circumferential residual stresses in the symmetrical positions of 0°–45° and 120°–180° are the smallest. By comparing and analyzing Figure 13a,b, we can find that the area distribution of the high stress area of the single-path straight-through welding method path-1 is relatively single and exists only within about 45° before the end point of welding. While the high stress area of the two-part welding sequence path-2 is more symmetrical and has a larger distribution area, there are large circumferential residual stresses at the welding start and end points of the rear welding side, but this welding sequence (path-2) also reduces the circumferential residual stress on the first welding side, which is at the 105°–180° area of path-2. The circumferential residual stress is significantly lower than path-1.

Figure 13c shows the distribution of the circumferential residual stresses along the defined path for Case 7–9 (path-3). From the figure, it can be found that the maximum value of the residual stress is 328 MPa, which occurs at the position of the 162.5° region of Case 7 (interlayer temperature of 150 °C). We also found an interesting phenomenon that the circumferential residual stress in the 0°–90° and 180°–270° regions is lower than the circumferential residual stress in the 90°–180° and 270°–0° regions, the reason is that the residual stress in the first section of the weld’s residual stress is significantly smaller than the second section of the weld. For the welding sequence path-3, the positions of 0°, 90°, 180° and 270° are the start and end points of the weld, all of which have experienced four thermal cycles, but the circumferential residual stresses at the 90° and 270° positions are lower than those at the 0° and 180° positions. This may be due to the fact that the previous weld will be reheated when the subsequent process is performed, so the residual stress caused by the previous welding process will be significantly changed. If the heat input of the subsequent weld channel is large enough, the stress of the previous weld and its vicinity will be greatly offset because, during heating, the high temperature can sufficiently soften the area.

From Figure 13a–c, it can be found that the interlayer temperature mainly affects the maximum value of the residual stress and the overall magnitude of the residual stress. When the interlayer temperature is 150 °C or 300 °C, the peak value of the residual stress is larger. The welding sequence has little effect on the maximum value of residual stress. The welding sequence mainly affects the distribution of residual stress and is a decisive factor affecting residual stress. By adjusting the welding sequence, not only can the residual stress distribution area be changed, but the residual stress in a specific area can also be reduced. Figure 13 also shows that the weld on the rear weld side largely changes the residual stresses within and near the first few welds, and the high stress areas are mostly concentrated at the weld on the rear weld side.

**Figure 14:**
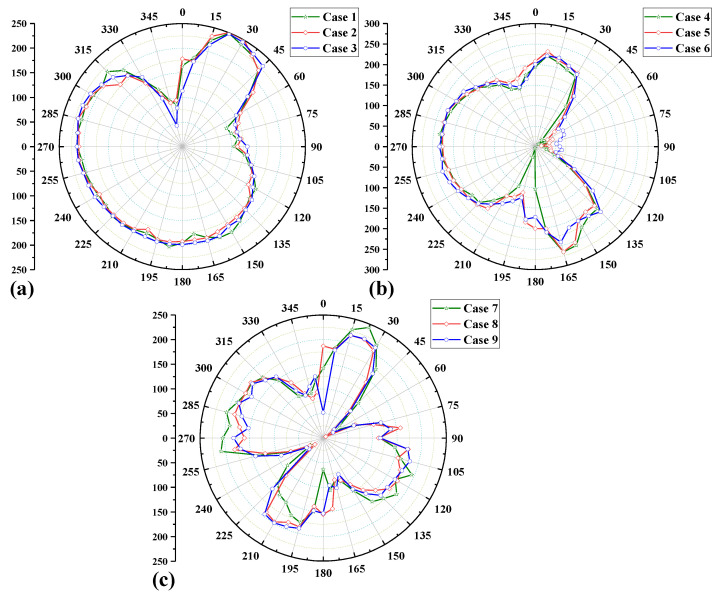
Axial residual stress distribution along path-4 for different welding cases. (**a**) Case 1–3 (welding sequence path-1) (**b**) Case 4–6 (welding sequence path-2) (**c**) Case 7–9 (welding sequence path-3).

Figure 14a–c compares the axial residual stress distribution along the defined path under nine simulation cases. Similar to the circumferential residual stress, the effect of interlayer temperature is small, while the difference in cases for different welding sequences is obvious. The maximum axial residual stress is 249 Mpa. Figure 14a shows the axial residual stress distribution of Case 1–3 (path-1); it can be seen that the peak residual stress still occurs under the welding Case 3. The axial residual stresses near the 0° position (welding start/end point) vary quite significantly, which is very similar to the conclusions drawn above for the circumferential residual stresses. At the same time, it can be seen that the area with higher axial residual stress of the welding sequence path-1 is distributed at the position of 0°–45°, which is on the right side of the welding starting point of the weld. The area with higher circumferential residual stress of path-1 is mainly on the left side of the end point of the weld. The last welded seam mainly affects the distribution of the high stress area of the circumferential residual stress, while the most initially welded seam mainly affects the distribution of the high stress area of the axial residual stress.

Figure 14b compares the axial residual stress distribution of Case 4–6 (path-2) along the defined path. The maximum value of the axial residual stress is 266 Mpa, which appears in Case 5. The axial residual stress in the range of 0°–180° experienced a change trend of increasing–decreasing–increasing, and the areas where the residual stress increased were mainly distributed near the welding start and end positions. However, it is reduced near the 90° area in the middle of the weld. This is because 0° and 180° positions are the welding start and end points of the welding sequence path-2, respectively, and the residual stress near the end of the weld is slightly larger than that near the starting point of welding. Furthermore, we found that the residual stress distribution at the position of the 180°–360° region (left side of Figure 14b) has good continuity, but the overall residual stress value is larger than the right side.

Figure 14c shows the axial residual stress distribution of Case 7–8 (path-3) along the defined path. It can be seen that the axial residual stress distribution of path-3 is discontinuous, and there are multiple fluctuations. The maximum value of the residual stress is 243 Mpa, which appears in Case 1. However, the overall value of the residual stress in the defined path of path-3 is smaller than that of path-1 and path-2. The start point and end point of the welding seam of path-3 are at the same position, and they are located at the positions of 0°, 90°, 180° and 270°, respectively. The axial residual stresses near these four locations are lower, and higher axial residual stresses are distributed at the weld seam in the weld steady state, which is the opposite trend to the distribution of the circumferential residual stresses in path-3.

From the Figure 13 and Figure 14, we can know that the value of the circumferential residual stress along the defined path is usually lager than the value of the axial residual stress. Setting a reasonable range of interlayer temperature can effectively reduce the peak value of the residual stress of the weld. The welding sequence directly determines the distribution of the welding residual stress, and there will always be large fluctuations in the residual stress near the welding start/end point. The above mainly analyzes the residual stress distribution at the weld, and the following will further analyze the residual stress on both sides of the weld. In addition, the maximum values of the circumferential and axial residual stresses occur in the welding cases with interlayer temperatures of 150 °C or 300 °C. Therefore, we choose the welding cases with interlayer temperatures of 200 °C and analyze the residual stresses on the weldolet side and the branch side at different cross-sections.

### 4.4. Effect of Welding Sequence on Residual Stress

**Figure 15:**
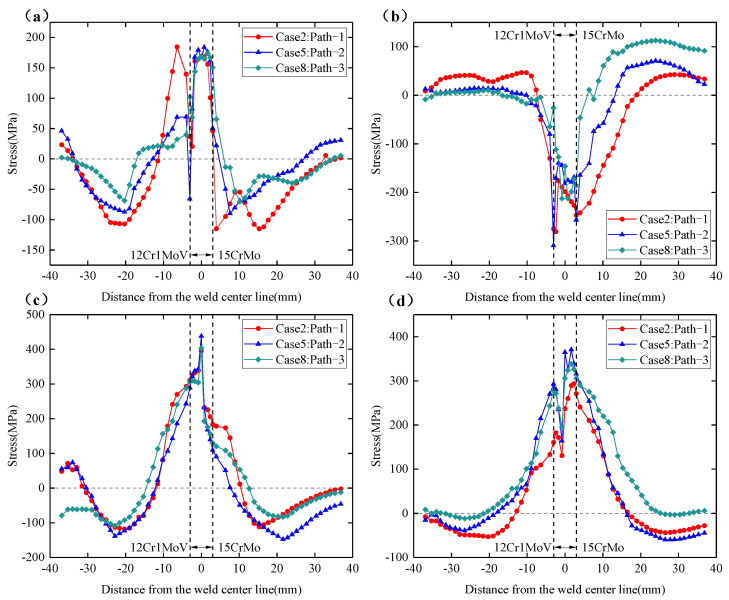
Circumferential and axial residual stress distribution of Case 2, Case 5 and Case 8 at 180° section. (**a**) Circumferential residual stress on the outer surface. (**b**) Axial residual stress on the outer surface. (**c**) Circumferential residual stress on the inner surface. (**d**) Axial residual stress on the inner surface.

From the above conclusions, it can be seen that the number of thermal cycles experienced by the three welding sequences at 0° position is the same, and the distribution of residual stresses is very similar, so the following analysis focuses on the distribution of residual stresses at 180°, 90° and 270° sections. Figure 15a–d shows the circumferential and axial residual stress distributions on the inner and outer surfaces at the 180° section. It can be seen from the figure that, although path-1 experienced only two thermal cycles at the 180° position, the overall residual stress distribution trend for path-1 is similar to that of path-2 and path-3. The residual stresses in the three welding sequences did not show a significant magnitude relationship. From Figure 15a,b, the circumferential residual stress on the outer surface has experienced tensile stress-compressive stress-tensile stress from the center of the weld to both sides of the weldolet and branch pipe, while the axial residual stresses on the outer surface experienced compressive-tensile stresses. The residual stress distribution on both sides of the weld is not symmetrical. There is a very large stress gradient at the fusion line on both sides of the weld, and the peak value of the axial residual stress reaches 310 Mpa on the 12Cr1MoV steel side. This is due to the large differences in the thermophysical properties of dissimilar materials, and it is also a very obvious feature of dissimilar steel welding. The thermal expansion coefficients of 12Cr1MoV steel and 15CrMo steel are different, and the expansion and contraction of the two materials are different during the welding heating and cooling processes, which ultimately causes the residual stress gradient on both sides of the weld to be larger and the distribution asymmetrical.

Figure 15c,d shows the circumferential and axial residual stress distributions on the inner surface at the 180° section. The circumferential residual stress on the inner surface has a similar distribution trend to that on the outer surface, while the axial residual stress on the inner surface has an opposite direction to that on the outer surface. It can also be found that the circumferential and axial residual stress values at the inner surface of the weld are significantly higher than the outer surface, the circumferential residual stress peak on the inner surface is about 425 Mpa, and the axial residual stress peak is about 370 Mpa. This shows that the residual stress of the 12Cr1MoV/15CrMo dissimilar steel welded joint is more obvious in the bottom weld. At the 180° section, the adjustment of the welding sequence caused the change in the peak value and distribution trend of the welding residual stress, but it is impossible to judge which welding sequence is better.

**Figure 16:**
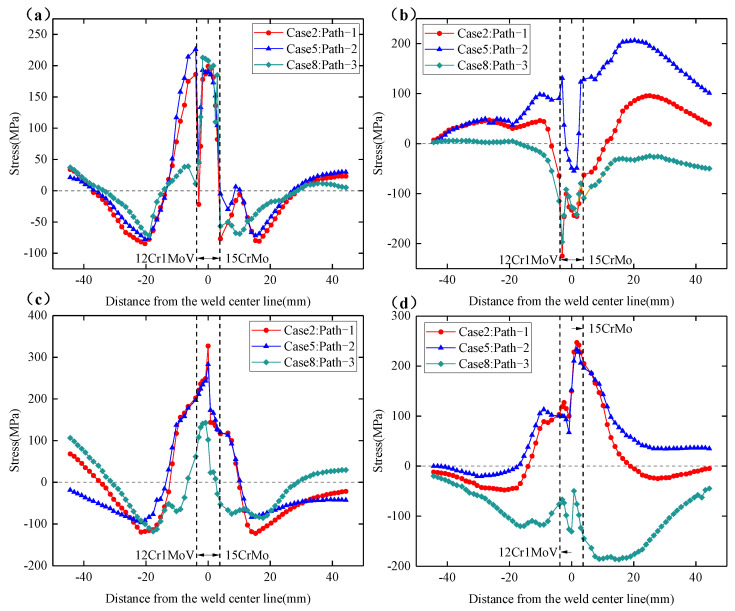
Circumferential and axial residual stress distribution of Case 2, Case 5 and Case 8 at 90° section. (**a**) Circumferential residual stress on the outer surface. (**b**) Axial residual stress on the outer surface. (**c**) Circumferential residual stress on the inner surface. (**d**) Axial residual stress on the inner surface.

Figure 16a–d shows the circumferential and axial residual stress distributions on the inner and outer surfaces at the 90° section. From Figure 16a,b, it can be found that the circumferential residual stress distribution trend of the outer surface at the 90° section and the 180° section is very similar, but the axial residual stress on the outer surface is quite different. At the 90° section, the distribution of residual stress reflects a certain regularity with the change in welding sequence. The circumferential residual stresses at the outer surface welds of the three welding sequences are relatively close, but the residual stress distributions on the weldolet side and the branch pipe side show obvious differences. Among them, the overall distribution of the circumferential residual stress on the weldolet side and the branch pipe side of path-3 is smaller than that of path-1 and path-2. It can also be seen that there is no significant difference in the axial residual stress on the outer surface of path-1 at 90° and 180°, but the direction of the axial residual stress on the outer surface of path-2 and path-3 has changed. At the 90° section, the axial residual stresses on the weldolet side and the branch pipe side of path-2 all show tensile stress characteristics, and path-3 also mainly shows tensile stress characteristics. The axial residual stress on the outer surface has an obvious magnitude relationship. The magnitude of the axial residual stress on the weldolet side and the branch side is: path-3 < path-1 < path-2. This may be because path-3 has experienced more thermal cycles at the 90° position. The multiple heating process is equivalent to the tempering process, which helps to reduce the residual stress inside the weld.

Figure 16c,d shows the distribution of the circumferential and axial residual stresses on the inner surface. It can be seen that the residual stress values near the weld on the inner surface are higher than those on the outer surface, which is consistent with the distribution of residual stress in the 180° section. Compared with the 180° section, the most obvious change in the internal surface residual stress at the 90° section is still path-3. The overall distribution of the circumferential residual stress on the inner surface of path-3 is smaller than the other two welding sequences, and the peak residual stress at the weld is also smaller, only 146 MPa. The axial residual stresses on the inner surface are all characterized by compressive stresses in the 90° section, which is exactly the opposite of the 180° section. It can also be found from the figure that the axial residual stresses at the inner surface welds of path-3 are smaller than those of path-1 and path-2, but they are larger on the weldolet side and the branch side than path-1 and path-2, and the axial residual stresses on the branch side are somewhat larger than those on the seat side. The 180° position is the arc ending point of path-2, which is also the arc starting point/ending point of path-3. Both path-2 and path-3 have experienced four thermal cycles at 180°, but the axial residual stress distribution pattern of the inner and outer surfaces of path-2 at 180° is completely different from that of path-3. This indicates that the number of thermal cycles is not a single factor affecting the distribution of residual stresses; the location of the weld start/end point distribution can also affect the distribution of residual stresses.

**Figure 17:**
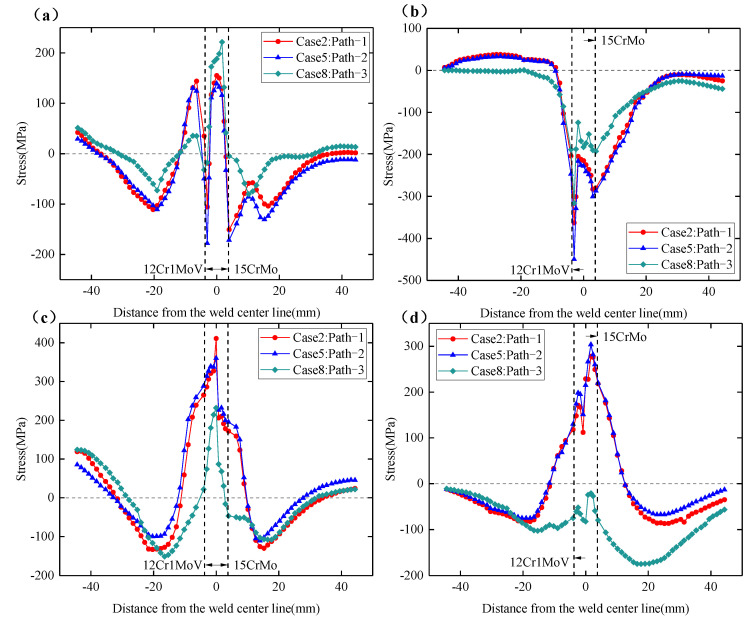
Circumferential and axial residual stress distribution of Case 2, Case 5 and Case 8 at 270° section. (**a**) Circumferential residual stress on the outer surface. (**b**) Axial residual stress on the outer surface. (**c**) Circumferential residual stress on the inner surface. (**d**) Axial residual stress on the inner surface.

Figure 17a–d shows the residual stress distribution on the inner and outer surfaces at the 270° section. The 270° position is the symmetry point of the 90° position, and the overall trend of the circumferential residual stress distribution on the inner and outer surfaces of these two sections is similar, but the axial residual stress on the inner and outer surfaces is different. The axial residual stresses on the outer surfaces of path-1 and path-2 at the 90° section are mainly tensile stresses, while at the 270° section they are mainly compressive stresses, and the peak of compressive stresses are much higher than those at the 90° section. This may be because the 270° position belongs to the rear weld side of path-1 and path-2, and the residual stresses on the rear weld side tend to be larger. The axial residual stress distribution in the 90° and 270° sections of path-3 is similar because path-3 is a segmented jump welding method. The first welding area is the 0°–90° and 180°–270° area position. By comparing the residual stress distribution at the 270° section of the three welding sequences, we can find that the overall distribution of circumferential and axial residual stresses on the outer surface of path-3 is smaller than path-1 and path-2, and the inner surface has the same pattern of circumferential residual stresses. The axial residual stress at the weld on the inner surface of path-3 is much smaller than path-1 and path-2, but the axial residual stress on the weldolet and branch pipe is larger than path-1 and path-2, and eventually tends to 0 MPa.

## 5. Conclusions

In this study, through numerical simulation and experimental verification, the effect of different interlayer temperatures and welding sequences on the temperature field and stress field of 12Cr1MoV/15CrMo dissimilar metal arc welding joints were studied. The conclusions are summarized as follows:

(1) In this study, the Case 2 (interlayer temperature of 300 °C, welding sequence path-1) model was selected to verify the residual stress and transient temperature. The simulation results are in good agreement with the experimental results, which verifies the reliability of the experimental model.
(2) The transient temperature distribution results show that the differences in the thermophysical properties of the materials during welding of dissimilar steels lead to an asymmetric distribution of the transient temperature field about the center of the weld. The high temperature range of the 15CrMo steel side is larger than that of the 12Cr1MoV steel side, and the heat-affected zone is relatively wider.
(3) The circumferential and axial residual stress distribution on both sides of the weldolet–branch pipe is not symmetrical, and there are always large fluctuations and stress gradients in the weld and the area near the weld. There is always a peak residual stress at the weld fusion line on both sides of the weld.
(4) The interlayer temperature of the weld has little effect on the distribution pattern of the residual stresses, which mainly affect the peak residual stresses. The peak residual stress along the defined path occurs more often at interlayer temperatures of 150 °C or 300 °C.
(5) The welding sequence is a decisive factor affecting the residual stress. By adjusting the welding sequence, not only can the residual stress distribution area be changed, but also the residual stress in specific areas can be reduced. The post-welded seam largely changes the residual stresses near the first weld, and the high stress areas are mostly concentrated at the post-weld.
(6) The number and position distribution of the start/end point of the weld also effect the distribution of residual stress, and the distribution trend of circumferential residual stress and axial residual stress on the outer surface is opposite. The overall residual stress distribution at the 90° section is smaller than the other three sections.
(7) At the 0° and 180° sections, the residual stress distribution of path-1, path-2 and path-3 are very similar, and the circumferential and axial residual stresses on the inner surface are larger than those on the outer surface. However, the residual stresses on the inner and outer surfaces of the segmental jump welding method path-3 are smaller than those of path-1 and path-2 in the 90° and 270° sections as a whole. The residual stresses reach a minimum in the 90° section. It can be concluded that path-3 is more suitable for the welding of weldolet–branch pipe dissimilar steel joints.

## Figures and Tables

**Table 1:** Welding methods and parameters.

Welding Method	Levels	Filling Material	Welding Wire (bar) Diameter/mm	Current I/A	Voltage U/V	Welding Speed mm/s	Gas Flow
GTAW	1	R30	2.5	80~110	15~20	1	8 L/min
SMAW	2	R307	3.2	90~120	20~24	1.3	

**Table 2:** Mechanical and thermal properties of weld metals.

Weld Metals	Melting Point/°C	Steel Plate Thickness/mm	Tensile Strength/Mpa	Yield Strength/Mpa	Elongation	Temperature/°C	Impact Energy/J
12Cr1MoV	1520 °C	6~60	440~590	≥245	≥19	≥20	≥47
15CrMo	1500 °C	6~60	450~590	≥295	≥19	≥20	≥31

**Table 3:** Welding simulation cases.

Case	Welding Sequence	Preheating Temperature/°C	Interlayer Temperature/°C
Case 1	Path-1	150	150
Case 2	Path-1	200	200
Case 3	Path-1	300	300
Case 4	Path-2	150	150
Case 5	Path-2	200	200
Case 6	Path-2	300	300
Case 7	Path-3	150	150
Case 8	Path-3	200	200
Case 9	Path-3	300	300

## Data Availability

The data presented in this study are available upon request from the corresponding author. The data are not publicly available due to the requirements of related projects.
